# Editorial: Rising stars in aquatic physiology: 2022

**DOI:** 10.3389/fphys.2022.1081961

**Published:** 2022-11-10

**Authors:** E. Gisbert, D. C. Moreira, M. T. Mozanzadeh, K. Lu

**Affiliations:** ^1^ Aquaculture Program, Institut de Recerca i Tecnología Agroalimentaries (IRTA), La Rápita, Spain; ^2^ Faculty of Medicine, University of Brasilia, Brasília, Brazil; ^3^ South Iran Aquaculture Research Centre, Iranian Fisheries Science Institute (IFSRI), Ahwaz, Iran; ^4^ Fisheries College, Jimei University, Xiamen, China

**Keywords:** physiology, elasmobranch, teleost, stress, longevity, mollusc

Promoting and visualizing cutting-edge research from young generations is the cornerstone of our academic environment. Under this scenario, this Research Topic aims gathering innovative research studies on aquatic physiology, studies that range from ecological field studies focused on how ocean acidification might affect cleaning fish services in reefs (Paula et al.) to molecular biology studies dedicated to the characterization of genes involved in longevity in two molluscan species (Xu et al.) and cortisol receptors expression in a teleostean fish (Vallejos-Vidal et al.). Additionally, a fourth manuscript has been published in this Research Topic, a very interesting study describing how parental metabolite provisioning is directed to offspring (both spermatozoa and developing embryos) in an elasmobranch lecithotrophic viviparous species (Wosnick et al.).

Among the field studies in the current Research Topic, Paula et al. evaluated how climate change, and in particular ocean acidification (OA), may affect cleaning stations in coral reefs since cleaner fish motivation to interact with clients decreased with OA. Thus, client fishes in cleaning stations must deal with the physiological impacts of OA together with the stress induced by ectoparasitism, which may ultimately compromise their physiological fitness and performance. Authors found that the lack of access to cleaning services lead to more physiological tolerance to parasite infection, whereas, independent of access to cleaners, high CO_2_ lowered fish fitness, although this was not exacerbated by parasite infection. Such observation is of relevance since cleaner fish provide an essential ecological service to their clients (cooperative cleaning interactions) by controlling their ectoparasite loads through direct removal of ectoparasites from their surfaces and reducing infection rates. The information provided by this study highlights the importance of integrative ecological studies and provided hints about the impact of climate change on complex fish interactions in communities.

The second study in this Research Topic dealing with field-collected fish was authored by Wosnick et al. The authors studied the metabolite provisioning to gametes and developing embryos through parental matrices in the viviparous Brazilian guitarfish (*Pseudobatos horkelii*). This species is of special relevance because a considerable fraction of parental energy reserves is reallocated for gamete production and embryo development, making its reproductive period particularly vulnerable. In this sense, results from this study revealed that metabolite provisioning to sperm and developing embryos through parental matrices were influenced by female and male diets during the reproductive period. Authors also revealed that, while seminal fluid metabolites may present a protective function, it is possible that nutritional provision also occurs. Furthermore, this study highlighted that studies on energy mobilization directed to offspring through parental fluids may support unravelling metabolic dynamics during the reproduction stage, as well as providing support for stress physiology studies to evaluate the indirect effects of parental allostatic overload in both sperm and developing embryos.

The other two research articles featured in the current Research Topic dealt with molecular biology studies on molluscans (Xu et al.) and teleost fish (Vallejos-Vidal et al.). In particular, the study by Xu et al. investigated the function of phosphatase and tensin homolog deleted on chromosome ten (PTEN) gene in two closely related *Argopecten* spp. scallops with significantly distinct life spans. The relevance of such study is that PTEN is involved in the regulation of longevity through several pathways, such as regulation of energy availability, genomic integrity, and oxidative damage caused by reactive oxygen species. Under this scenario, authors confirmed that starvation stress decreased metabolic activity, which might reduce lipid metabolism, protein biosynthesis, protein hydrolysis, cellular respiration, and increased glucose xenobiotic activity; thus, indicating that PTEN played an important role in the physiological response of the scallops to nutrient availability. Different responses in RNA levels of PTEN between both scallop species indicated different capacities to adapt to different environments, resulting in changes in long-term adaptations and providing insight in the role of this protein. Additionally, the findings may support the selection of scallops’ strains with long lifespan and thus larger size, which is of special relevance considering the economic important of this group of bivalve species.

The last study included in this Research Topic, by Vallejos-Vidal et al., focused on the description of the gene expression profile of mineralocorticoid receptor (*mr*) and glucocorticoid receptors (*gr1* and *gr2*) linked to their function as cortisol receptors and mediators of the physiological stress response in gilthead seabream (*Sparus aurata*). Cortisol is the main corticosteroid and plays an important role in many physiological areas including growth, immunoregulation, and energy maintenance. Vallejos-Vidal et al. evaluated the transcriptomic regulation of *mr*, *gr1*, and *gr2*, as well as that of several genes involved in the host’s immune response, in different mucosal tissues like the skin, gills, anterior gut after stressing fish by air exposure (3 min). Authors confirmed that *gr2* and *mr* had higher affinity for cortisol than *gr1*, allowing possible the ligand-receptor interaction at a very low cortisol levels, whereas *gr1* responsible for the recognition of cortisol at high plasmatic levels. Additionally, the authors also reported that stress modified the transcriptomic response of immune genes in a time- and tissue-dependent manner, information that it is considered of relevance because of the importance of stress response in farmed fish species. Overall, this Research Topic highlights the relevance of the field and laboratory studies conducted on diverse species and the contribution of young researchers to the development of aquatic physiology.

